# 

**Published:** 1901-06-29

**Authors:** 


### NOTICE TO CORRESPONDENTS.

All MSS., Letters, Books for Review, and other matters intended for the Editor should be addressed The Editor, "The Hospital," The Hospital Building, 28 & 29 Southampton Street, Strand, W.C.

The Editor cannot undertake to return rejected MS., even when accompanied by stamped directed envelope.

**Notice to Advertisers and Subscribers.**

All Advertisements, Orders for Copies of The Hospital, and Business Communications should be addressed to **THE MANAGER (Not to the Editor),** The Hospital, 28 & 29 Southampton Street, Strand, London, W.C.

The Cost of Subscription, post free, is as follows:—Per week, 2½d.; per quarter, 2s. 8d.; per half-year, 5s. 3d.; per annum, 10s. 6d. Foreign:—Per annum, 15s. 2d., payable, in all cases, in advance.

**NOTICE.—Vol. XXIX. of "The Hospital" (October 1900, to March, 1901), in handsome cloth binding is now on sale at the Office of this Journal, price, 7s. 6d. Cases for binding the Numbers contained in this Volume 1s. 6d. Index to Vol. XXIX., 2½d., post free.**

**The Monthly Part for July will be ready on July 1st. Price 6d., by post 8d.**

### SCRAPS AND GLEANINGS.

THE total amount collected on Hospital Sunday in Sheffield was £2,042.

THE building and equipment of the Totnes Cottage Hospital have cost £2,220, towards which £2,146 has been received.

THERE were 8,807 attendances during last year at the Glasgow Ear Hospital, being an average of five attendances for each of the 1,658 patients.

THE treasurer of the Rochdale Infirmary has received a cheque in payment of a legacy of £50, less duty, bequeathed to the institution by the late Mrs. Emma Wood, of 140 Drake Street.

THE cost of the new ward and additions to the Alexandra Hospital at Woodhall Spa is about £2,000. This increased accommodation enables 30 patients to be treated.

DURING 1900 the sum of £2,[illegible]94 was contributed by the employees of 384 firms to the Bradford Joint Hospital Fund; the total amount of contributions received for this Fund was £13,000, while the expenditure was £16,000.

LADY LLANGATTOCK has conceived the excellent idea of a children's memorial to Queen Victoria, which is to take the form of providing a children's cot in the Newport County Hospital (Monmouthshire), the cost of which is to be met by the children contributing a penny or more according to circumstances

THE enlargements to be made at the Quarrier Sanatoria for Consumption give ten additional beds. The total cost of the Sanatoria, including these enlargements, will be £11,395, all of which has been given by anonymous friends. These sanatoria are for the use of females only from any part of the country; 80 patients can be treated at one time, and £8,000 per annum is needed for maintenance.

As a result of the fortnight's inquiry that the honorary staff of the Royal Portsmouth Hospital have been holding in connection with the alleged abuse of the out-patient department, the committee have come to the conclusion that a large proportion of the persons who obtain tickets are in a position to pay for assistance from private practitioners. Subscribers are asked to exercise the greatest care in the issue of tickets.

### THE STUDENT OF MEDICINE.

### APPOINTMENTS.

**James W. Allen**, M.B.Glasg., appointed Physician Glasgow Royal Infirmary. **G. A. Barr**, L.R.C.P.Lond., M.R.C.S.Eng., appointed Medical Officer to the Isle of Wight Union Workhouse. **H. S. Elworthy**, F.R.C.S., appointed Casualty Officer to the Great Northern Central Hospital. **E. F. Eyre**, L.R.C.P., L.R.C.S.Edin., appointed Medical Officer to the Islington Parish Workhouse, Cornwallis Road.

**F. Harcourt Gervis,** M.D.Brux., M.R.C.S., L.R.C.P.Lond., appointed Medical Officer to the Hampstead Provident Dispensary (West Hampstead Branch). **F. D. Hayman,** M.R.C.S.Eng., appointed Medical Officer of Health for Ararat, Victoria. **H. E. Hewitt,** M.B.Lond., appointed District Medical Officer of the Eastbourne Union. **E. B. Hulbert,** M.D., appointed Assistant Medical Superintendent to the St. Marylebone Infirmary. **F. H. Jacob,** M.B.Lond., appointed Honorary Assistant Physician to the Nottingham General Hospital. **E. Harries Jones,** M.B., C.M.Edin., appointed Ophthalmic Surgeon to the Northampton General Infirmary. **P. K. Lewis,** M.R.C.S.Eng., appointed Medical Officer of the Bromyard Union Workhouse. **Beatrice F. Lovibond,** M.B., appointed House-Surgeon at the Leyton, Walthamstow, and Wanstead Children's and General Hospital, Orford Road, Walthamstow. **G. F. McCleary,** M.D., D.P.H.Camb., appointed Medical Officer of Health for the Metropolitan Borough of Battersea. **Joseph Maguire,** L.R.C.P., L.R.C.S.I., appointed Medical Officer for the Ballinrobe Dispensary District. **Owen Paget,** M.B., B.S.Cantab., appointed Medical Officer of Health for Freemantle, Western Australia. **Herbert George Parker,** F.R.C.S., L.R.C.P.Edin., appointed Honorary Ophthalmic Surgeon to the Bolton Infirmary and Dispensary. **A. M. N. Pringle,** M.B., C.M.Edin., appointed Medical Officer of Health to the Manchester Port Sanitary Authority. **G. W. Pritchett,** M.R.C.S.Eng., L.R.C.P.Lond., appointed Assistant Medical Officer to the North-West London Hospital, Kentish Town. **J. C. R. Robinson,** M.R.C.S., L.R.C.P.Lond., appointed District Medical Officer of the Depwode Union. **William Rose,** M.B., F.R.C.S., appointed Consulting Surgeon to the Great Eastern Railway. **Harold Douglas Singer,** M.D.Lond., M.R.C.S., L.R.C.P., appointed Senior House-Physician to the National Hospital for the Paralysed and Epileptic, Queen Square. **J. W. Smith,** M.B., M.S.Edin., F.R.C.S.Eng., appointed Honorary Assistant Surgeon to the Manchester Royal Infirmary. **J. P. Sparks,** M.D.Durh., appointed District Medical Officer of the Tynemouth Union. **J. Sparks,** M.D.Durh., appointed Medical Officer of Health for the Whitley and Monkseaton Urban District Council. **F. W. Stansfield,** M.D.Vict., D.P.H.Camb., appointed District Medical Officer of the Reading Union. **Jas. A. Stephen,** M.A., M.B., D.P.H.Aberd., appointed Medical Officer for the Parish of St. Andrew's-Lhanbryd, Elgin. **Edgar Stevenson,** M.D.Aberd., appointed Surgeon to the Liverpool Eye and Ear Infirmary. **R. D. A. Stone,** L.R.C.P., L.R.C.S.I., appointed District Medical Officer of the Devizes Union. **J. Taylor,** M.D.Aberd., appointed Certifying Factory Surgeon for the Elgin District. **Wm. Atkin Thompson,** M.R.C.S.Eng., L.R.C.P.Lond., appointed House Surgeon to the Windsor and Eton Royal Infirmary and Dispensary. **Kathleen Olga Vaughan,** M.B.Lond., appointed Resident Medical Officer to the Royal Aberdeen Hospital for Sick Children. **W. M. Wanklyn,** M.R.C.S., L.R.C.P.Lond., appointed Medical Superintendent to the Westmorland Sanatorium for Consumption. **W. B. Warrington,** M.D., M.R.C.P., appointed Physician to the David Lewis Northern Hospital, Liverpool. **J. W. Watson,** M.B., C.M.Aberd., appointed Parochial Medical Officer for New Spynie. **P. A. Wedgwood,** L.R.C.P., L.R.C.S.Edin., L.F.P.&S.Glasg., appointed District Medical Officer of Green Hammerton and the Great Ouseburn Union, and Public Vaccinator, Green Hammerton District, Great Ouseburn Union. **Cresswell F. White,** M.B., C.M.Aberd., L.S.A.Lond., appointed District Medical Officer for the Templecombe District of the Wincanton Union. **J. H. Wigham,** L.R.C.P., L.R.C.S.Edin., appointed Medical Officer of Health for the Borough of Southmolton. **Egerton H. Williams,** M.R.C.S.Eng., L.R.C.P.Lond., D.P.H.Camb., M.D.Brux., appointed Medical Superintendent of the City Hospitals, Sheffield. **W. B. Herepath Wood,** M.B.Lond., M.R.C.S.Eng., appointed Medical Officer for the new Consumptive Sanatoria at Delamere Forest.

King's College Hospital. — The following resident medical appointments have been made at this hospital:— House Physicians: **W. L. Stuart,** M.R.C.S., L.R.C.P.; **C. G. Bulteil,** M.R.C.S., L.R.C.P.; **F. Carle,** M.R.C.S., L.R.C.P. House Surgeons: **T. M. Jefferiss,** M.R.C.S., L.R.C.P.; **R. Jaques,** M.R.C.S., L.R.C.P.; **E. A. Sanders,** M.R.C.S., L.R.C.P. House Accoucheurs: **W. W. Maxwell,** M.R.C.S., L.R.C.P.; **J. A. MacLeod,** M.R.C.S., L.R.C.P.

